# Comparative Evaluation of Computational Methods for Validating Housekeeping Gene RT-qPCR Data in 3T3-L1 Cells

**DOI:** 10.3390/biomedicines13082036

**Published:** 2025-08-21

**Authors:** Zhenya Ivanova, Natalia Grigorova, Valeria Petrova, Ekaterina Vachkova, Georgi Beev

**Affiliations:** 1 Department of Pharmacology, Animal Physiology, Biochemistry and Chemistry, Faculty of Veterinary Medicine, Trakia University, 6000 Stara Zagora, Bulgaria; zhenya.ivanova.12@trakia-uni.bg (Z.I.); valeriya.petrova@trakia-uni.bg (V.P.); ekaterina.vachkova@trakia-uni.bg (E.V.); 2 Department of Biochemistry, Microbiology and Physics, Faculty of Agriculture, Trakia University, 6000 Stara Zagora, Bulgaria; georgi.beev@trakia-uni.bg

**Keywords:** RT-qPCR, normalization, reference genes, 3T3-L1, *L. paracasei*, HPRT

## Abstract

**Background:** Postbiotics with anti-adipogenic properties can significantly modify adipocyte metabolism by influencing key cellular pathways involved in lipid accumulation. In preliminary in vitro studies, it is essential to monitor various cellular and subcellular variables, including gene expression and protein synthesis potential, through RT-qPCR analysis. It is also crucial to select internal controls carefully and evaluate their stability for effective normalization and accurate interpretation of the results. **Methods:** In this study, we assessed the stability of six commonly used housekeeping genes: GAPDH, Actb, HPRT, HMBS, 18S, and 36B4. We analyzed their variability in mature 3T3-L1 adipocytes treated with supernatants from newly isolated *Lacticaseibacillus paracasei* strains. Our analysis combined classical statistical methods, a ∆Ct analysis, and software algorithms such as geNorm, NormFinder, BestKeeper, and RefFinder. **Results:** Our stepwise, multiparameter strategy for selecting reference genes led to the exclusion of Actb and 18S as the most variable reference genes. We identified HPRT as the most stable internal control. Additionally, HPRT and HMBS emerged as a stable pair, while the recommended triplet of genes for reliable normalization consists of HPRT, 36B4, and HMBS. **Conclusions:** The widely used putative genes in similar studies—GAPDH and Actb—did not confirm their presumed stability, which once again emphasizes the need for experimental validation of internal controls to increase the accuracy and reliability of gene expression. Combining a unique biological model—postbiotic-treated adipocytes—with multiple algorithms integrated into a single workflow allows us to provide a methodological template applicable to similar nutritional and metabolic research settings.

## 1. Introduction

Probiotics play a key role in maintaining overall health. Current studies have focused on the relationship between intestinal microbiota composition and obesity-related metabolic disorders [1,2,3,4]. Members of the genera *Lactobacillus* and *Bifidobacterium* additionally can provoke hypolipidemia and hypoglycemia, influencing key physiological processes in white adipose tissue [5,6,7,8]. These effects are mediated by biologically active soluble factors—so-called postbiotics—secreted by live bacteria and absorbed in the intestine. Reaching peripheral tissues, they modulate signaling pathways related to metabolic balance [9,10]. Postbiotics exhibit most of the beneficial effects of probiotics, including their antimicrobial, antioxidant, anti-obesogenic, and immunomodulatory properties, and excluding some of their negative characteristics [9,11,12,13,14]. The existing intestinal flora exercises a minimal influence on them and, once absorbed, they launch a more targeted action in easily controlled doses and remain stable during storage and transportation. Postbiotics are particularly important in people with compromised immunity, highlighting the need for modern nutrigenomic approaches to move towards personalized strategies [15,16,17,18,19,20].

Due to the significant strain specificity in the metabolic profile, there is a real scientific and practical need to identify new strains with substantial health effects. *Lacticaseibacillus paracasei* (*L. paracasei*) is a well-known lactic acid bacterium, widely used in the food industry [21,22]. Some strains exhibit antimicrobial and anti-inflammatory and stress-response modulating activity [23,24], with their efficiency being strictly strain-specific. However, insufficiently studied probiotics can lead to adverse effects, such as diarrhea, bacterial translocation, or even bacteremia in risk groups [24,25]. Therefore, it is crucial to identify and characterize new, safe, and functional strains. The autochthonous *L. paracasei* strains isolated from unique ecological niches have attracted particular scientific interest [22,26]. Their preliminary screening is often based on in vitro molecular analysis of key genes’ regulatory mechanisms and expression patterns. When assessing eventual probiotic and postbiotic modulation of fat metabolism, the 3T3-L1 cell line, derived from mouse preadipocytes, is considered a universal model for monitoring adipocyte differentiation, glucose transport, lipid metabolism, and insulin sensitivity, prior to the shift to in vivo experiments [27,28,29]. These cells have been used for studying anti-adipogenic *Lactobacillus*-mediated effects in numerous recent investigations [8,30,31,32,33,34].

In these initial studies, molecular technologies, such as the reverse transcription quantitative polymerase chain reaction (RT-qPCR), provide a powerful tool for precise gene expression analysis [35]. Reliabile RT-qPCR results require stable internal standards (reference genes) to correct sample-to-sample RNA and reverse transcription variations [36,37,38]. Postbiotics with antiadipogenic properties, such as *L*. *paracasei* metabolites, can alter adipocyte metabolism and affect the expression of widely used housekeeping genes (HKGs) such as glyceraldehyde-3-phosphate dehydrogenase (GAPDH) and actin beta (Actb), which are involved in glycolysis and cytoskeletal structure [39,40].

The Minimum Information for Publication of Quantitative Real-Time PCR Experiments (MIQE) guidelines recommend at least two stable reference genes validated by algorithms such as NormFinder, geNorm, and BestKeeper [41,42,43,44,45]. However, recent publications investigating the impact of *L. paracasei* on adipocyte metabolism have often relied on a single reference gene without stability assessment or a solid rationale for its selection [10,46,47,48]. This poses a significant risk of acquiring unreliable RT-qPCR results, especially given the complex metabolic changes induced.

Therefore, the current study aimed to evaluate the expression stability of six commonly used HKGs (GAPDH, Actb, hypoxanthine-guanine phosphoribosyltransferase (HPRT), hydroxymethylbilane synthase (HMBS), 18S ribosomal RNA (18S), and ribosomal protein lateral stalk subunit P0 (36B4)) in mature 3T3-L1 adipocytes treated with supernatants from newly isolated *L. paracasei* strains (M2.1, C8, C15, and P4), applying the widely used mathematical algorithms geNorm, NormFinder, BestKeeper, and RefFinder, as well as classical ∆Ct and statistical data analyses.

## 2. Materials and Methods

### 2.1. Propagation and Adipogenic Induction of Preadipocytes

Cryopreserved 3T3-L1 mouse fibroblasts (CRL-3242, ATCC, Washington, DC, USA) were thawed and seeded in T75, following the manufacturer’s instructions for propagation. After two passages to obtain sufficient cell mass, cells were plated at a density of 1 × 10^4^/mL in 12 and 24-well plates for the subsequent experimental procedures. During the proliferation phase, the cells were grown in basal medium (BM) composed of high-glucose Dulbecco’s modified Eagle’s medium (DMEM, 4.5 g/L glucose with L-glutamine), (Sigma-Aldrich Chemie GmbH, St. Louis, MI, USA), 10% (*v*/*v*) fetal bovine serum (FBS) (Sigma-Aldrich Chemie GmbH), and 1% antibiotic solution (penicillin G, streptomycin and amphotericin B, Sigma-Aldrich Chemie GmbH). Cultures were maintained in a 95% humidity incubator at 37 °C and 5% CO_2_. Upon reaching 100% confluency, cells were growth-arrested for 24 h and treated for 48 h with adipogenic induction media (AIM) to induce adipogenesis. AIM was composed of DMEM, 4.5 g/L glucose, 10 µg/mL insulin (cell application, San Diego, CA, USA), 0.05 mM indomethacin (Sigma-Aldrich Chemie GmbH (Merck KGaA, Darmstadt, Germany), 0.1 mM 3-isobutyl-1-methylxanthine (Cayman Chemical, Ann Arbor, ML, USA), and 1 µM dexamethasone (Sigma-Aldrich, St. Louis, MO, USA). For seven days following induction, adipocytes were cultured in maintenance media (AMM) containing BM supplemented with 10 µg/mL insulin until they reached full maturity according to the differentiation protocol.

### 2.2. L. paracasei Cell-Free Supernatants Preparation and Application on Mature Adipocytes

The isolation of the autochthonous microorganisms *L. paracasei* strains M2.1, C8, C15, and P4, their identification, subsequent proliferation, and preparation of cell-free supernatants have been described previously [49]. Immediately before application, the pH of all supernatants was neutralized to pH 7 and applied for 48 h to already differentiated 3T3-L1 adipocytes. They were divided into six groups: untreated control (IC), treated with 10% *v*/*v* de Man, Rogosa, and Sharpe broth controls (MRS), and treated with 10% *v*/*v* supernatants from *L. paracasei* strains (M2.1, C8, C15, and P4). Each experimental group consisted of six independent culture wells (biological replicates) obtained from the same cell passage and cultured in parallel under identical conditions.

### 2.3. Intracellular Lipid Deposition Visualization

To confirm successful adipogenesis and visualize potential phenotypic differences between mature adipocytes from the various experimental groups, cells were stained with Oil Red O (Sigma-Aldrich, St. Louis, MO, USA) at the end of the experiment. The supernatant from each well was carefully removed, and the cells were washed three times with phosphate-buffered saline (PBS). The adipocytes were then fixed in 10% (*v*/*v*) neutral buffered formalin for 10 min, washed with isopropanol for 5 min, and stained for 30 min with a freshly prepared working solution of Oil Red O. Following staining, the wells were rinsed with PBS, and images were acquired using a Leica DMi1 Inverted Microscope equipped with a 5-megapixel-resolution camera.

### 2.4. Gene Expression Assays

Total mRNA was isolated from previously lysed adipocyte cells using the RNeasy Mini Lipid Tissue Kit (QIAGEN GmbH, Hilden, Germany), strictly following the manufacturer’s instructions. The purity and integrity of RNA samples were determined spectrophotometrically at 260 and 280 nm wavelengths using a Take3 Microvolume Plate of Synergy™ LX Multi-Mode Microplate Reader (BioTek Instruments, Inc., Santa Clara, CA, USA). The OD ratio in all samples was in the range of 1.9 to 2.1, which confirmed the sound quality of the obtained RNA. Then, cDNA was synthesized using a RevertAid First Strand cDNA Synthesis Kit (Thermo Scientific, Waltham, MA, USA); the RNA concentration was previously equalized across all samples to 1000 ng/µL. The subsequent RT-qPCR analyses were performed with KAPA SYBR^®^ fast qPCR Master Mix kit (QIAGEN Sciences, Inc.; Germantown, MD, USA). A SYBR Green-based two-step real-time PCR method was employed to evaluate gene expression using the PikoReal-Time PCR System (PikoReal, Thermo Scientific, Waltham, MA, USA). The PCR reaction was performed using the temperature program recommended by the manufacturer of the SYBR Green Real-Time PCR Master Mix.

### 2.5. Primer Design

Table 1 shows the primer sequences for qPCR of the tested HKGs. Most of the primers were designed using open-access data in NCBI software (www.ncbi.nlm.nih.gov, accessed on 20 October 2024) following the manufacturer’s instructions described in SYBR Green Master Mix. Only 18S sequences were previously designed by Arnhold et al. [50]. The specific primer pairs were further confirmed using the additional tools Primer-BLAST (NCBI) https://www.ncbi.nlm.nih.gov/tools/primer-blast (accessed on 21 October 2024), Primer 3 (https://primer3.ut.ee/, accessed on 21 October 2024), Primer 3plus (version: 3.2.5) (https://www.bioinformatics.nl/cgi-bin/primer3plus/primer3plus.cgi, accessed on 21 October 2024), and SerialCloner (version 2.6.1). The web-based software Primer 3 was also used to determine the product length. Then, the melting temperatures and predicted melting curves of the RT-qPCR products were analyzed using uMelt QuartzSM (Version 3.6.2) (https://www.dna-utah.org/umelt/quartz/um.php, accessed on 22 October 2024).

A temperature gradient was performed to determine the optimal annealing temperature for the primers before RT-qPCR analysis. The optimal annealing temperature for each primer pair was found to be very close to the commonly recommended value of 60 °C, with efficient amplification observed within the range of 58 to 61 °C.

The specificity of the amplification products was confirmed by gel electrophoresis and melting curve analysis, and the results can be provided upon request.

Reaction efficiency for each primer pair was calculated using the PicoReal software 2.2 based on standard curves generated from a 6-point 5-fold serial dilution of cDNA. Efficiencies between 90% and 110%, with correlation coefficients (R^2^) ≥ 0.99, were considered acceptable. The achieved results indicate efficiency values of the tested genes ranging between 98% and 99%.

### 2.6. Analysis of Expression Stability Among Selected HKGs

Four validation software tools, NormFinder (an Excel add-in, MS Office version 2016) [31], geNorm v3.5 [37], BestKeeper v1 [33], and RefFinder (https://www.ciidirsinaloa.com.mx/RefFinder-master/, accessed on 26 May 2025) [38], were applied to investigate the stability of candidate reference genes.

NormFinder was utilized through its original Excel-based macro. The software is available for download as an Excel add-in (.xla format) from the official NormFinder source. Relative expression quantities were generated using PicoReal Software 2.2 (Thermo Scientific) and imported directly into the algorithm to evaluate gene stability based on intra-group and inter-group variation–stability value (CV). There are no strictly defined maximum acceptable CV values, but a value below 0.15 is often considered indicative of good stability [42].

The relative expression levels of the analyzed HKGs were used to assess their stability using the Excel-based geNorm macro (geNorm v3.5, Windows/VBA, Ghent, Belgium: Center for Medical Genetics, Ghent University, https://genorm.cmgg.be/, accessed 20 May 2025). The algorithm calculated stability values (M) through pairwise comparisons of all genes with a threshold of M < 1 for heterogeneous samples, while M < 0.5 was preferred for homogeneous ones [43,51]. Lower M-values indicated greater stability [52].

The BestKeeper Excel-based tool (Munich, Germany:Technical University of Munich, Institute of Physiology, https://www.gene-quantification.de/bestkeeper.html, accessed on 19 May 2025) was used to analyse raw Ct values of the candidate reference genes and the target gene PPARγ. The software calculated descriptive statistics, including standard deviation (SD), coefficient of variation (CV), arithmetic and geometric means, minimum and maximum Ct values, and fold variation. Pearson correlation coefficients (r) were estimated between each reference gene and the BestKeeper index to assess internal consistency. A regression analysis was also performed to evaluate expression stability further [44]. In addition, a Pearson correlation analysis was performed between each candidate reference gene and the target gene PPARγ to measure potential co-regulation under the experimental conditions. The correlation coefficient (r) and corresponding *p*-value were extracted and reported from this analysis. The commonly suggested BestKeeper thresholds for reliability of the internal control include a standard deviation (SD) of less than 1, a coefficient of variation (CV) below 2%, and a Pearson correlation coefficient with the BestKeeper index (r) ≥ 0.9 (*p* < 0.05) [44,53].

RefFinder was used as an online tool (https://www.ciidirsinaloa.com.mx/RefFinder-master/ (accessed on 21 October 2024)) to integrate the results from all algorithms and provide a final ranking of the reference genes based on the geometric mean of their Ct-derived stability ranks [54].

A pairwise ΔCt-based stability analysis was evaluated manually in Microsoft Excel according to the method of Silver et al. [55]. The standard deviation of the ΔCt values across all samples was calculated for each pair of candidate reference genes. Genes with lower average standard deviations were considered more stable.

### 2.7. Statistical Analysis

Statistical analyses and figures visualization were performed using GraphPad Prism 10.5.0 (774) Software LLC (Boston, MA, USA) and Statistica version 10 (StatSoft Inc., 2011, Tulsa, OK, USA). Descriptive statistics were used to calculate the mean and standard deviation (SD) for ΔCt values, and the standard error of the mean (SEM) for raw Ct values. The results are shown in the figures as means ± SEM. The normality of data distribution was assessed using the Shapiro–Wilk test. For normally distributed data, one-way ANOVA and Student’s *t*-test were applied to assess intergroup differences in Ct values, while the non-parametric Mann–Whitney test was used for comparative analysis of SD, using the GraphPad Prism tool. A *p*-value less than 0.05 (*p* < 0.05) was considered statistically significant.

## 3. Results

### 3.1. Adipogenic Induction and LB Treatment

**Figure 1:**
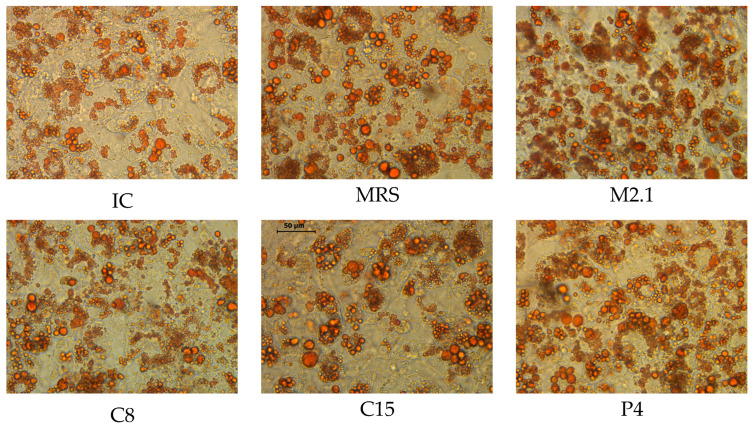
Morphology and intracellular neutral lipid accumulation in mature 3T3-L1 cells, stained with Oil Red O, after 48 h of 10% (*v*/*v*) cell-free supernatant treatments (20× magnification, bar = 50 µm). Images were acquired using a Leica DMi1 Inverted Microscope equipped with a 5-megapixel-resolution camera and the Leica Application Suite Core software 4.12.0 (Heerbrugg, Switzerland). Abbreviations: IC—mature non-treated cells; MRS—mature cells, treated with MRS broth; M2.1, C8, C15, and P4—mature cells, treated with supernatants from M2.1, C8, C15, or P4 strains.

The obtained microscopic images stained with Oil Red O proved the successful adipose differentiation (Figure 1). After 9 days of induction, followed by 48 h of treatment with supernatants of four *L. paracasei* strains and MRS, the cells displayed distinct morphological and phenotypic features characteristic of differentiated adipocytes, including the accumulation of numerous lipid droplets.

### 3.2. Expression Stability Assessment via Four Popular Algorithms

#### 3.2.1. NormFinder and geNorm

According to the average M-values evaluated by geNorm software v3.5, presented in Table 2, all of the genes cover the minimal requirements for homogeneous samples except 18S (M = 0.510)*,* which revealed itself as the least stable reference gene. HPRT is outlined as the most reliable HKG for further normalization, confirmed also by NormFinder analysis (Table 2).

#### 3.2.2. BestKeeper and RefFinder

In contrast to the previously discussed algorithms, which rely on relative expression quantities, BestKeeper and RefFinder evaluate gene stability using raw Ct values. According to the descriptive statistical analysis provided by BestKeeper (Table 3), the genes HPRT, HMBS, 36B4, GAPDH, and Actb meet the commonly accepted criteria for reference gene stability, mentioned above. Based on these parameters, the genes were ranked as follows: HPRT was the most stable, followed by HMBS, 36B4, GAPDH, and Actb. The gene 18S slightly exceeded the commonly accepted CV threshold (CV = 2.13%).

Data from the BestKeeper pairwise correlation and regression statistics were further evaluated to assess the candidate reference gene variability (Table 4). Genes with higher Pearson’s correlation coefficient (r) values, closer to 1.0, are regarded as more stably expressed under the experimental conditions [44]. The r values ranged from 0.48 (18S) to 0.80 (36B4), with 36B4 and HPRT showing the highest correlations (r = 0.80 and r = 0.79, respectively).

The equally strong determination coefficients (r^2^ = 0.63) for both HPRT and 36B4 support their suitability as reliable internal controls. Moreover, 36B4 and HPRT exhibited slope values closest to 1, with values of 1.00 and 0.99, respectively, indicating that their expression levels are proportional to the BestKeeper index. HMBS, 18S, and GAPDH showed moderate deviation, while Actb demonstrated a more significant divergence with a slope of 1.31. The higher slope and standard error for Actb (±0.316) suggest variability in its expression. All *p*-values were below 0.001, confirming the statistical significance of the observed correlations (Table 4).

Additionally, a pairwise correlation analysis of each HKG vs. a target gene, PPARγ, was performed. A strong correlation indicates that the same biological factors could influence a reference gene as the target gene. Among the genes analyzed, only Actb demonstrated a strong and statistically significant positive correlation with PPARγ, with an r value of 0.759 and a *p*-value of 0.001 (Table 5).

**Figure 2:**
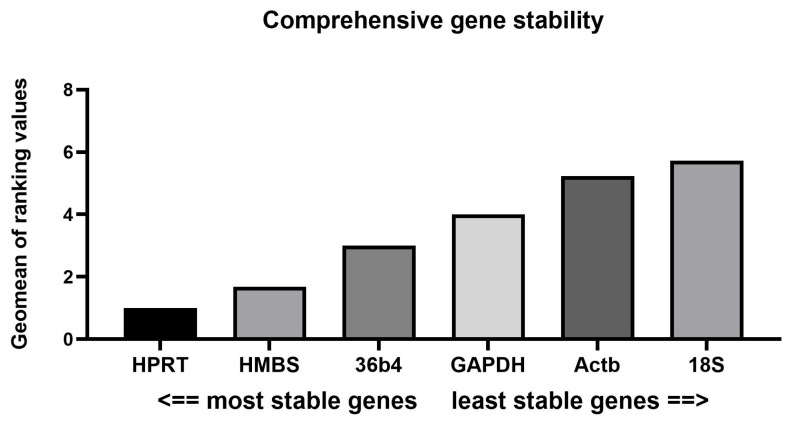
Comprehensive ranking of candidate reference genes calculated via the RefFinder tool. Data were obtained from six experimental groups with six biological replicates each (*n* = 36 per gene). The geometric mean of ranking values integrates stability scores from geNorm, NormFinder, BestKeeper, and the ΔCt method, with lower values indicating higher expression stability. Visualization was performed in GraphPad Prism (v.10).

In confirmation of the results obtained so far, the ReFinder tool also highlighted HPRT as the most stable gene (geomean rank = 1), followed by HMBS (1.68) and 36B4 (3). These genes demonstrated consistent performance across the applied evaluation methods and thus could be recommended for reliable normalization (Figure 2).

### 3.3. Pairwise ΔCt Analysis

The stability of the candidate reference genes—HPRT, 18S, 36B4, GAPDH, HMBS, and Actb—was also evaluated using the pairwise ΔCt method described by Silver et al. [55]. The analysis revealed that several gene combinations involving HPRT, 36B4, and HMBS exhibited the lowest variability, suggesting stable expression across the tested samples. Conversely, 18S and Actb were associated with greater ΔCt variation, indicating their lower reliability as internal controls (Table 6).

### 3.4. Inter-Group Statistical Analysis of Raw Ct Values for Reference Gene Evaluation

**Figure 3:**
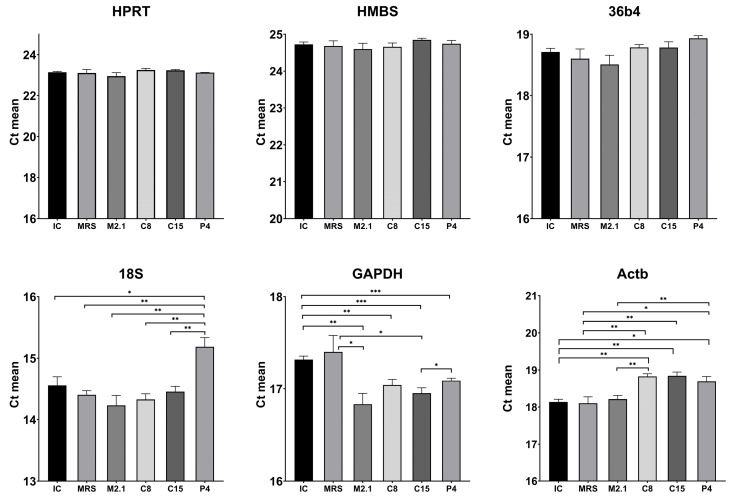
Inter-group statistical comparison of raw Ct values for selected reference genes (HPRT, HMBS, 36B4, 18S, GAPDH, and Actb) in mature 3T3-L1 adipocytes treated with 10% (*v*/*v*) cell-free supernatants from *L. paracasei* strains. Abbreviations: IC—untreated, induced control; MRS—induced control, treated with MRS; M2.1, C8, C15, P4—experimental groups, induced and treated with supernatants for 48 h. Data are presented as mean ± SEM of six biological replicates per group (*n* = 6). Statistical analyses and visualization were performed in GraphPad Prism (v.10). Inter-group variability was first assessed by one-way ANOVA, and genes showing statistically significant differences were further analyzed using Student’s *t*-test to confirm the significance level, as follows: * *p* < 0.05; ** *p* < 0.01; *** *p* < 0.001.

To further support the findings, we compared the mean Ct values of each individual HKG across the experimental groups (Figure 3). A statistical analysis using Tukey’s post hoc test revealed no significant differences and only minimal variation in Ct values for the genes 36B4, HPRT, and HMBS. In contrast, the genes 18S, GAPDH, and particularly Actb showed significantly greater variability between groups. These results confirm the pairwise ΔCt analysis and suggest that 36B4, HPRT, and HMBS are more reliable endogenous controls for normalizing gene expression.

## 4. Discussion

According to the principles of the three Rs—Replacement, Reduction, and Refinement—established for the ethical use of animals in biomedical and pharmaceutical research, each experiment is expected to minimize the need for animal models [56,57]. Thus, the initial step in studying the effects of a new food supplement, such as the supernatants from potential probiotics, would ideally involve a preliminary in vitro investigation using a suitable cell line aligned with the study’s objectives [58,59].

This approach facilitates the rapid screening of a wide range of molecular markers, allowing researchers to investigate the effects of different doses, administration, and other experimental parameters [60]. The simultaneous monitoring of multiple variables at the cellular and subcellular level, often in a highly dynamic cell system, such as differentiating mature adipocytes, establishing variations in gene expression patterns, and reporting the cell’s potential for protein synthesis. This is achieved through RT-qPCR analysis, which can detect even subtle changes in mRNA levels within a small sample volume over a very short period [61,62].

RT-qPCR is one of the most sensitive methods for measuring gene expression. However, it is prone to errors if approached or analyzed incorrectly. To ensure accurate measurement, it is essential to standardize and adapt several preanalytical steps, including sampling, RNA extraction and evaluation, optimization of reverse transcription, precise selection and testing of primers, as well as fine-tuning of the PCR reaction [63,64]. To unify the criteria and better comparability of the results obtained, in 2009, Bustin et al. [41] introduced standards for design, method of work, and description of the methods used for highly reliable publications, known as the MIQE guidelines, which we have adhered to in the present study.

The high precision and amplification efficiency of RT-qPCR are absolutely necessary but not sufficient to ensure the representativeness and repeatability of the obtained results. Nevertheless, an incorrect approach to analyzing the obtained results may misinterpret the target gene’s expression profile [65].

Quantifying RT-qPCR data is usually performed via absolute and relative methods [66]. In many nutrigenomic studies, it is usual to compare changes in the target gene(s) against an internal control, which is known as the relative method. Several mathematical models are applied for the relative quantification of gene expression, the most widespread being the delta delta Ct method (∆∆Ct) [67,68]. This method compares the differences in Ct values between the target and a reference gene (ΔCt) in both experimental and control groups, or so-called normalization of the target gene. The difference between these ΔCt values (ΔΔCt) is then used to calculate the relative gene expression. Therefore, selecting stable internal controls with an optimal efficiency of the RT-qPCR reaction is crucial for the reliability and relevance of RT-qPCR results [65,69].

In practice, however, such ideally stable HKGs are the exception, especially in cells undergoing dynamic physiological and metabolic changes. Actb or GAPDH is commonly cited in the literature as a single reference gene in in vitro studies investigating the effects of *Lactobacillus* strains, including *L. paracasei*, on the 3T3-L1 cells [31,46,47,48]. However, these studies frequently fail to provide experimental validation of the stability, amplification efficiency, or independence of these genes from particular study conditions. This raises concerns about the results’ validity and highlights the need for empirical confirmation of the reference gene choice.

Stability assessment of suitable genes should be conducted using statistical methods and tools such as geNorm, NormFinder, BestKeeper, and RefFinder. Supplementary Table S1 presents a summary of our analyses’ results as ranks. Most of the implemented approaches of stability evaluation pointed out the HPRT as the most stable reference gene under the tested experimental conditions.

However, according to the MIQE guidelines, it is not advisable to normalize data using only one internal control gene, as no gene remains stable under all experimental conditions. It is recommended to use at least two, and optimally three or more validated HKGs, the expression of which has been proven stable in the specific biological model [43,52]. Vandesompele et al. [52] also emphasize the importance of using at least three stable reference genes and introduce an improved ΔΔCt model, in which normalization is performed by calculating the geometric mean Ct values of the selected internal controls.

In our study, the stability values of the tested HKGs were relatively close. At first glance, all fell within the acceptable limits according to the stability guidelines of the respective algorithms. However, referring back to the MIQE requirements, abundant reference genes should be excluded if there is a significant difference in expression levels compared to the target gene. When there is such a significant difference (more than 5 Ct_s_), the reference gene’s amplification enters the exponential phase much earlier than that of the gene of interest, potentially compromising the sensitivity and accuracy of detecting actual expression changes.

To determine whether the internal control genes meet the specified criteria, we selected the highly expressed gene PPARγ as a specific target. PPARγ is a master regulator of adipogenesis and plays a crucial role in regulating lipid metabolism, insulin sensitivity, and the inflammatory status of mature adipocytes. This makes it a classic and widely used marker in studies evaluating potential hypolipidemic supplements’ effects and metabolic modulators [70,71].

The average Ct values are shown in Supplementary Table S1 The 18S gene exhibited the earliest expression, with a difference of more than 7 Ct units when compared to PPARγ. Therefore, 18S should be excluded from the normalization panel to avoid potential sensitivity loss during normalization. Additionally, the results of the BestKeeper analysis indicate that the intra-group variation for 18S exceeds the acceptable threshold of 2%, and the gene does not demonstrate a significant correlation with the BestKeeper index. The BestKeeper software v1 analyzes not only the internal consistency between the candidate reference genes but also the correlation of each of them with the overall index and with the target gene [44], which allows the detection of potential coincidences in expression trends.

It is important to note that the correlation coefficient of 18S with the BestKeeper index is 0.48, indicating that 18S does not correlate with the panel of internal controls. Moreover, it has a similar correlation with the BestKeeper index to that of the target gene, PPARγ (r = 0.44). While the moderate correlation with the BestKeeper index may not be critical on its own, the high inter-group variability among the internal controls increases the risk of misinterpreting the biological effects. This is especially true if any of the HKGs correlate with the expression of the target gene, as this would suggest that they are involved in the cellular response and are not neutral.

Such correlation with PPARγ (r = 0.759, *p* < 0.001) was observed in Actb gene expression, as shown in a gene-specific BestKeeper analysis. It appears that the effects exerted by the *L. paracasei* strains we investigated on mature 3T3-L1 adipocytes also involve remodeling of the actin cytoskeleton—a key event in adipogenesis that leads to PPARγ activation through mechanisms involving MKL1 and RhoA–ROCK signaling [72]. This finding contradicts a key criterion for selecting a reference gene: the expression of the reference gene should not correlate with that of the target gene to ensure reliable normalization and doubts about its role as a neutral internal control in this experiment. In accordance, the initial BestKeeper regression analysis of Actb revealed a high slope of 1.31, which further supports this conclusion since a slope greater than 1 indicates that the reference genes do not behave independently of the target gene [44].

To further confirm the selection of internal controls, we analyzed the intergroup variations in the expression of each gene using classical statistical methods. According to the generally accepted standard, ideal HKGs should maintain stable expression independent of experimental conditions [73,74,75]. Therefore, no statistically significant changes in expression between the different groups should be detected. A lack of significant differences between the groups was found only in HPRT, 36B4, and HMBS, highlighting their stability across experimental conditions.

Almeida-Oliveira et al. [76] point out that using multiple software tools simultaneously to validate internal control genes often leads to conflicting results, making it hard to identify a reliable and stable internal control. However, a clear and sensible ranking of these genes can be achieved by applying a systematic, step-by-step approach that considers all key stability criteria and gradually excludes variable genes.

In the current study, we employed a stepwise and multiparameter approach to select reference genes, which led to the well-founded exclusion of Actb and 18S due to their high variability. Our analysis clearly identified HPRT as the most stable reference gene. Additionally, HPRT and HMBS were found to be stable, while the recommended triplet of genes for reliable normalization included HPRT, 36B4, and HMBS. This reference gene set could serve as a useful starting point for validating housekeeping genes in similar in vitro studies based on mature adipocytes.

It is important to emphasize that developing and implementing such a methodological framework for selecting reference genes is consistent with current recommendations for standardization and transparency in gene expression analyses. Such approaches increase the reproducibility and reliability of the results, which are essential for interpreting molecular changes in biomedical models.

## 5. Conclusions

Our study highlights the need to carefully select internal controls in gene expression analysis in mature 3T3-L1 adipocytes under specific treatment with supernatants from newly isolated *L. paracasei* strains, a biological context that has not been previously investigated in this type of analysis. Based on the combined results of five independent algorithms and ∆Ct and inter-group statistical analysis of the raw Ct data from the RT_PCR reaction, HPRT was identified as the most stable gene, and the panel of HPRT, 36B4, and HMBS is recommended as a reliable combination for normalization in expression studies of similar nutritional supplements.

The widely used reference genes in similar studies—GAPDH and Actb—did not confirm their presumed stability. This again emphasizes the need for experimental validation of internal controls against specific conditions to increase the accuracy and reliability of RT-qPCR gene expression analyses.

Integration of several stability assessment algorithms into a single coherent workflow and its application to a unique metabolic model provided a ready-to-use validation framework for future nutrigenomic and postbiotic research.

## Figures and Tables

**Table 1:** Primer sequences and description of mouse housekeeping and target genes used in RT-qPCR.

Abbreviation	Full Name	Forward	Reverse	Product Length
36B4	Ribosomal protein, large, P0	TTATAACCCTGAAGTGCTCGAC	CGCTTGTACCCATTGATGATG	147
HPRT	Hypoxanthine-guanine phosphoribosyl transferase	ACAGGCCAGACTTTGTTGGA	ACTTGCGCTCATCTTAGGCT	150
Actb	Actin, beta	CCTCTATGCCAACACAGTGC	GTACTCCTGCTTGCTGATCC	211
HMBS	Hydroxymethylbilane synthase	CCTGAAGGATGTGCCTACCA	CCACTCGAATCACCCTCATCT	175
GAPDH	Glyceraldehyde-3-phosphate dehydrogenase	AAATGGTGAAGGTCGGTGTG	TGAATTTGCCGTGAGTGGAG	583
18S	18S ribosomal RNA	ATGCGGCGGCGTTATTCC	GCTATCAATCTGTCAATCCTGTC	204
PPARγ	Peroxisome proliferator-activated receptor gamma, transcript var. 2	AGGGCGATCTTGACAGGAAA	CGAAACTGGCACCCTTGAAA	164

**Table 2:** Stability values and ranking of reference genes by geNorm and NormFinder.

Gene Abbr.	Stability M-Value (geNorm)	Ranking	Stability Value (NormFinder)	Ranking
36B4	0.325	3	0.082	2
HPRT	0.299	1	0.071	1
Actb	0.452	5	0.202	5
HMBS	0.324	2	0.109	3
GAPDH	0.402	4	0.181	4
18S	0.510	6	0.215	6

**Table 3:** Descriptive statistics of the candidate reference genes obtained through the BestKeeper algorithm.

	18S	36B4	GAPDH	HMBS	HPRT	Actb
n	36	36	36	36	36	36
geo Mean [CP]	14.52	18.72	17.10	24.71	23.14	18.46
ar Mean [CP]	14.52	18.72	17.11	24.71	23.14	18.47
min [CP]	13.58	18.03	16.34	23.91	22.11	17.30
max [CP]	15.56	19.21	18.01	25.11	23.745	19.34
std dev [±CP]	0.31	0.20	0.21	0.19	0.16	0.33
CV [% CP]	2.13	1.08	1.25	0.77	0.70	1.80
min [x-fold]	−73.63	−24.12	−33.87	−38.69	−113.1	−211.3
max [x-fold]	119.8	9.39	62.82	6.23	16.44	54.91
std dev [±x-fold]	4.13	2.53	2.67	2.40	2.11	4.56

**Table 4:** BestKeeper pairwise correlation and regression analysis of candidate reference genes.

Regression Analysis: HKG vs. BestKeeper						
	18S	36B4	GAPDH	HMBS	HPRT	Actb
coeff. of corr. [r]	0.48	0.80	0.57	0.74	0.79	0.67
coeff. of det. [r^2^]	0.23	0.63	0.33	0.54	0.63	0.45
intercept [CP]	−3.64	−0.41	2.06	7.98	4.16	−6.64
slope [CP]	0.95	1.00	0.79	0.87	0.99	1.31
SE [CP]	±0.374	±0.165	±0.246	±0.175	±0.167	±0.316
*p*-value	0.00	0.00	0.00	0.00	0.00	0.00
Power [x-fold]	78.00	99.01	36.89	55.28	94.92	411.45

**Table 5:** Pairwise Pearson correlation coefficients (r) and *p*-values between candidate reference genes (18S, HPRT, HMBS, 36B4, GAPDH, Actb) and PPARγ based on BestKeeper analysis.

	18S	HPRT	HMBS	36B4	GAPDH	Actb
PPARγ coeff. of correlation. [r]	−0.130	0.463	0.342	0.512	−0.002	0.759
PPARγ *p*-value	0.452	0.004	0.041	0.001	0.992	0.001

**Table 6:** Pairwise ΔCt-based stability analysis of candidate HKGs according to the method of Silver et al. [55].

Gene Names	Mean Δ Ct	Std.Dev.	Mean STD. Dev. *
HPRT/18S	8.61	0.50	
HPRT/36B4	4.42	0.21	
HPRT/GAPDH	6.03	0.24	
HPRT/HMBS	1.57	0.17	
HPRT/Actb	4.67	0.34	0.29 ^a^
18S/36B4	4.20	0.48	
18S/GAPDH	2.58	0.51	
18S/HMBS	10.18	0.47	
18S/Actb	3.94	0.58	
18S/HPRT	8.61	0.50	0.51 ^b^
36B4/GAPDH	1.61	0.29	
36B4/HMBS	5.99	0.24	
36B4/Actb	0.32	0.24	
36B4/HPRT	4.42	0.21	
36B4/18S	4.20	0.48	0.29 ^a^
GAPDH/HMBS	7.60	0.28	
GAPDH/Actb	1.36	0.51	
GAPDH/HPRT	6.03	0.24	
GAPDH/18S	2.58	0.51	
GAPDH/36B4	1.61	0.29	0.37 ^ab^
HMBS/Actb	6.24	0.38	
HMBS/HPRT	1.57	0.17	
HMBS/18S	10.18	0.47	
HMBS/36B4	5.99	0.24	
HMBS/GAPDH	7.60	0.28	0.31 ^a^
Actb/HPRT	4.67	0.34	
Actb/18S/	3.94	0.58	
Actb/36B4	0.32	0.24	
Actb/GAPDH	1.36	0.51	
Actb/HMBS	6.24	0.38	0.41 ^ab^

* Alphabetic indices indicate statistically significant differences in mean SD value between Housekeeping genes, evaluated by Mann–Whitney nonparametric test; *p* < 0.05.

## Data Availability

The datasets generated for this study are available from the corresponding author upon request.

## Supplementary Materials

The following supporting information can be downloaded at: https://www.mdpi.com/article/10.3390/biomedicines13082036/s1, Table S1. Summary of stability ranking of candidate reference genes across multiple analytical methods and their average Ct values.

## Abbreviations

HKG(s)	Housekeeping gene(s)
RT-qPCR	Reverse transcription quantitative polymerase chain reaction
RNA	Ribonucleic acid
cDNA	Complementary deoxyribonucleic acid
36B4	Ribosomal protein, large, P0
HPRT	Hypoxanthine-guanine phosphoribosyl transferase
Actb	Actin, beta
HMBS	Hydroxymethylbilane synthase
GAPDH	Glyceraldehyde-3-phosphate dehydrogenase
18S	18S ribosomal RNA
PPARγ	Peroxisome proliferator-activated receptor gamma, transcript variant 2
*L. paracasei*	*Lacticaseibacillus paracasei*
MIQE	Minimum Information for Publication of Quantitative Real-Time PCR Experiments
DMEM	Dulbecco’s modified Eagle’s medium
FBS	Fetal bovine serum
PBS	Phosphate-buffered saline
BM	Basal medium
AIM	Adipogenic induction media
AMM	Maintenance media
MRS	de Man, Rogosa, and Sharpe broth-treated induced control group
IC	Untreated, induced control group
M2.1, C8, C15, P4	Experimental group of induced adipocytes treated with 10% *v*/*v* supernatants from the respective *L. paracasei* strain
SD	Standard deviation
CV	Coefficient of variation
r	Pearson correlation coefficient
Ct	Cycle threshold
∆Ct	delta Ct
∆∆Ct	delta delta Ct
