# Supplementary material for: Comparative Evaluation of Computational Methods for Validating Housekeeping Gene RT-qPCR Data in 3T3-L1 Cells
Source: Biomedicines. 2025 Aug 21;13(8):2036. doi: 10.3390/biomedicines13082036 (PMC12383485; doi:10.3390/biomedicines13082036)
Supplement: Supplementary file 1 [file biomedicines-13-02036-s001.zip › biomedicines-3820027-supplementary.pdf]

Table S1. Summary of stability ranking of candidate reference genes across multiple analytical methods and their average Ct values.

	Mean±SD	ΔCt Method Rank	NormFinder Rank	geNorm Rank	BestKeeper (statistic) Rank	BestKeeper (regression) Rank	BestKeeper gene-specific analysis	ReFinder (consensus) Rank	Tukey Intergroup Variation
**n**	**36**	**36**	**36**	**36**	**36**	**36**	**36**	**36**	**6 gr. /n=6**
HPRT	23.14 ± 0.27	1	1	1	1	2	Moderate	1	NS
HMBS	24.71 ± 0.25	3	3	2	2	3	Weak	2	NS
36B4	18.72 ±0.27	2	2	3	3	1	Moderate	3	NS
GAPDH	17.11 ± 0.29	4	4	4	4	5	Non	4	S
Actb	18.47 ± 0.42	5	5	5	6	4	**Strong**	5	S
18S	14.52 ± 0.42	6	6	6	5	5	Non	6	S
PPARγ	21.77 ± 0.57	-	-	-	-	-	-	-	S

Mean Ct ± SD – mean Ct value and standard deviation for each HKG across all samples

ΔCt Method Rank – stability ranking using the comparative ΔCt method

NormFinder Rank – based on combined intra- and inter-group variation (stability value)

geNorm Rank – based on M value (average pairwise variation)

BestKeeper (statistic) Rank – based on average rank of standard deviation (SD), coefficient of variation (CV%) and SD ± x-fold variation of Ct values

BestKeeper (regression) Rank – based on average rank of Pearson correlation coefficient (r), coefficient of determination (R^2), and slope of regression from linear regression against the BestKeeper Index

BestKeer gene-specific analysis –based on Pearson's r between Ct of each candidate reference gene and PPARγ expression; Abbr.: Non ($r < 0.20$), Weak ($0.20 \le r < 0.40$), Moderate ($0.40 \le r < 0.75$), Strong ($r \ge 0.75$, $p>0.05$).

ReFinder (consensus) Rank – summary ranking based on geometric mean of individual gene ranks from ΔCt, geNorm, NormFinder, and BestKeeper

Tukey Intergroup Variation – based on statistical differences in Ct values across groups (p-values) evaluated by Tukey's post hoc test. Abbr.: NS (non-significant differences, $p>0.05$), Significant (minimum three significant differences between groups, $p<0.05$); (**6 gr. /n=6 -** 6 groups with 6 biological replicates each)
